# The Attitudes of Patients with Cardiovascular Diseases towards Online Exercise with the Mobile Monitoring of Their Health-Related Vital Signs

**DOI:** 10.3390/sports12020047

**Published:** 2024-02-01

**Authors:** Apostolia Ntovoli, Maria Anifanti, Georgia Koukouvou, Alexandros Mitropoulos, Evangelia Kouidi, Kostas Alexandris

**Affiliations:** 1 Department of Physical Education, Sports Sciences Frederick University, Nicosia 3080, Cyprus; 2 Laboratory of Sport Medicine, Department of Physical Education and Sport Science, Aristotle University of Thessaloniki, 57000 Thermi, Greecekouidi@phed.auth.gr (E.K.); kalexand@phed.auth.gr (K.A.)

**Keywords:** online exercise programs, patients with cardiovascular diseases, online monitoring

## Abstract

The health care cost of cardiovascular diseases (CVD) in the EU is estimated to be today over 282 billion euros. It is well documented today that exercise training is one of the main strategies for secondary disease prevention and the follow-up integration of these patients. This study aimed to examine patients’ attitudes towards online exercise with mobile monitoring of their vital signs. More specifically, the research objectives were as follows: (a) to examine patients’ attitudes and expectations of online exercise, (b) cluster patients in high- and low-attitude groups and examine their intention to participate in online exercise, and (c) to examine age and gender differences in terms of their intention to exercise online. The final goal of this project was to develop a real application that could be of use to patients and professionals. Data were collected from fifty patients in the city of Thessaloniki, Greece. The results revealed that most patients were positive about exercising online if the programs were perceived as fun and, especially, safe. The use of an online monitoring application with the distant supervision of health professionals could both motivate them and strengthen their feeling of safety.

## 1. Introduction

CVD of the heart and blood vessels include coronary heart disease, heart failure, high blood pressure, stroke, peripheral vascular disease, rheumatic heart disease, and other conditions. The rapid increase in CVD is a major medical and social problem worldwide, as it is associated with increased morbidity and mortality. As CVD are the most common cause of death worldwide, they account for approximately 17.9 million deaths each year [1]. Correspondingly in Europe, CVD are responsible for approximately 3.9 million deaths per year (45% of total deaths) and for the highest rate of premature death for people under the age of 65 (1 in every 3 deaths). As life expectancy in developed countries increases, so does the prevalence of cardiovascular disease [2]. Based on the latest statistics, after two decades of gains, Greece’s life expectancy at birth experienced a sharp decrease of 1.5 years between 2019 and 2021 because of the COVID-19 pandemic. With a slight rebound in 2022, it returned to the 2012 level of 80.7 years, which is equal to the EU average. Stroke, ischemic heart disease, and lung cancer are the leading causes of death [3].

CVD are estimated to cost the EU EUR 282 billion annually across the 27 European Union countries, representing a cost of EUR 630 per EU citizen. CVD-related costs were estimated using country-specific national data on morbidity, mortality, and health, social, and informal care. Health and long-term care accounts for EUR 155 billion (55%), forming 11% of EU health expenditure. Productivity losses accounted for 17% (EUR 48 billion), whereas informal care costs were EUR 79 billion (28%). Coronary heart disease accounted for 27% (EUR 77 billion), and cerebrovascular diseases accounted for 27% (EUR 76 billion) of CVD costs [4].

About 1–2% of national health care budgets are spent on the treatment of cardiovascular disease. Considering that more than 60% of these costs are related to hospitalization, the need for an early diagnosis and better regulation of increased cardiovascular risk factors becomes apparent [5].

The increase in the incidence of CVD in developed countries is attributed to factors of daily lifestyle, mainly related to lack of physical activity, unhealthy eating habits, which bring obesity, but also increased stress, and smoking. Concerning Greece, the latest Eurostat showed that it is the second country in the inactivity ranking in the EE after Bulgaria. A total of 67% of the adult population do not participate in any form of physical activity. Identifying individuals at the highest cardiovascular risk who may experience deterioration and/or dysregulation of their clinical condition and ensuring appropriate treatment can prevent early deaths and frequent hospitalizations.

This study is part of a bigger funded project aiming to develop an online application for monitoring the online exercise of patients with CVD and their health-related vital signs. The purpose of this paper is to examine patients’ attitudes toward online exercise as a first step to examining the demand for such an application and its attributes. This study contributes to the literature by reporting, for the first time, results of the attitudes of patients with CVD towards online exercise and about the development of a monitoring mobile application, using attitude theory (the tricomponent attitude model). These results are also of applied importance since they can guide future synergies among health and exercise professionals in their attempt to increase the safe participation of individuals with CVD in exercise programs.

## 2. Theoretical Background

### 2.1. Exercise and Cardiovascular Diseases

Primary and secondary prevention to reduce CVD and adverse cardiovascular events is the primary goal of preventive medicine and health policy in developed countries. Trying to reintegrate patients into daily life after an ischemic heart attack is very difficult. In recent decades, several studies have proven the important role played by cardiovascular rehabilitation programs, mainly in the form of systematic exercise, in the prevention and treatment of CVD and the improvement of quality of life [6].

The beneficial effect of systematic physical activity on cardiovascular morbidity and mortality is widely accepted today, as well as on cardiovascular risk factors in patients with CVD. Regular exercise training provides both primary and secondary disease prevention of cardiovascular disease and is highly recommended in patients with coronary artery disease, who benefit the most [6]. Systematic physical activity has been found to contribute to a better regulation of blood pressure, show favorable effects on lipid and carbohydrate metabolism, offer anti-inflammatory benefits, improve the action of the autonomic nervous system in the heart, and exert a beneficial effect on endothelial function [7]. Thus, patients with chronic heart failure are encouraged to participate in exercise-based rehabilitation programs [8]. On the other hand, even in patients with low functional capacity, who are candidates for implantable cardioverter defibrillators, left-ventricular assist devices, cardiac resynchronization devices, and heart transplantations, both endurance and resistance exercises with low cardiovascular demand should be offered [9].

The exercise programs for patients with cardiovascular disease should be individualized according to the functional capacity and health problems of everyone, as well as be feasible, effective, and safe [8]. Considering that patients with CVD belong to vulnerable groups, especially in the case of pandemics such as COVID-19, it is recommended to avoid congregating and instead keep distance. Consequently, it is understood that patients cannot participate in group-organized exercise programs in cardiovascular rehabilitation centers. The implementation of suitable exercise programs at each patient’s home with the simultaneous monitoring of the patient’s vital signs (blood pressure, heart rate, oxygen saturation, and body temperature) with modern telemedicine applications is the appropriate solution for effective and safe exercise for patients with CVD [10].

### 2.2. Status of Cardiovascular Disease Management

Chronic heart diseases are one of the leading fields of application of mobile health (mHealth). In recent years, efforts have focused on providing applications that enable patients to monitor their cardiovascular health for the early detection of arrhythmias and the prevention of cardiovascular events. The global number of smartphone users was forecast to continuously increase between 2024 and 2028 by a total of 496.7 million users (+10.71 percent). After the fifth consecutive increasing year, the smartphone user base is estimated to reach 5.1 billion users and, therefore, reach a new peak in 2028 [11].

Many applications have been developed for the management of CVD, with significant functions (blood pressure level monitoring, charts, statistics, etc.). In 2017, hypertension and cardiovascular disease were by far the largest markets, with 1 billion cases each [12]. With the development of technology, smartwatches have now been given the ability to monitor the electrocardiogram and check vital signs. However, the majority of apps are lacking in quality, functionality, and important features for patients, such as reminders, updates on the disease, accuracy of results, and encouragement to adhere to medication [13,14,15,16], while, at the same time, they require attention to the interpretation—the understanding of the information they provide [17]. The minimization of the user’s participation in the data entry process (thus simplifying and facilitating his daily life) and detection—notification in case of a cardiovascular event—are considered important.

Therefore, the management of CVD through existing applications is a complex process, requiring high-level digital skills from the user/patient, only partially meeting his needs. This presents deficiencies in the combined utilization of the individual elements, because of which unreliable communication with the physician is often observed, which can lead to an incorrect treatment of the condition.

### 2.3. Research Objectives

The research objectives were to examine the following:

∘ Patients’ intention and attitudes to participate in online exercise;
∘ Patient’s expectations of online exercise;
∘ Cluster patients in groups with high and low attitudes and examine their intention to participate in online exercise;
∘ Age and gender differences in terms of patients’ intention to exercise online.

## 3. Methodology

Fifty patients (N = 50) with CVD participated in a quantitative study. The data were collected in 2021 by approaching hospital units and health professionals who provided us access to patients in the wider area of Thessaloniki (Greece). Accessibility was first asked for by the hospital unit administrators and the health professionals. All the patients participated on a voluntary base, after having given consent to participate. They were first informed about the goal and the outcome of the study. All the university ethics procedures were followed.

Snowball sampling was chosen as the most efficient sampling method. This non-probability sample is one of the limitations of this study. Since this is a non-random sample, it cannot be considered representative of the study population. The results, therefore, cannot be generalized with confidence. They, however, show some trends.

A questionnaire was developed using, as a base, the tri-component attitude theory [18]. This model proposes that attitude has three elements. The cognitive component includes an individual’s beliefs and knowledge about an attitude object; the affective component includes feelings towards this object; and the conative component includes an individual’s intentions toward this object. All three components were measured with this study’s scale. Bipolar adjectives were used to measure the affective (4 items) and cognitive (7 items) components of attitudes towards online exercise. Intentions to participate in the online program (conative component) were used with one item on a 5-point Likert scale. Expectations of online exercise participation were measured with six items on a 5-point Likert scale. Respondents were also asked to provide demographic information regarding their gender, marital status (single, married), age, and level of education, as well as information related to the chronic diseases they were dealing with. This demographic variable is typically one used in most of the studies conducted on sport and exercise consumer behavior [18]. The following three typical demographic questions were also used: gender, age, and occupation. These three variables are included in most sports behavior studies [18].

The age of the respondents was coded in nominal categories as follows: 18–25, 26–35, 36–45, and 46–65. Six levels of occupation were included as follows: private sector, public sector, entrepreneurs, housewives, university students, and others. The demographic characteristics of the sample are presented in Table 1. Most of the sample comprised men (72%). Regarding the age group, about two-thirds of the sample were between 26 and 35 (36%) and 46 and 65 (34%), followed by 36 and 45 (20%) and 18 and 25 (10%). Concerning occupational status, the majority were working in the public sector (44%), followed by entrepreneurs (18%), while the sample was almost balanced among those who were employed in the private sector (10%), the university students (10%), housewives (10%), and those who were employed in “other” occupations (8%). The medical profile of the sample is presented in Table 2.

### Statistical Analysis

Descriptive Statistics (% and mean scores) were used to analyze participants’ intentions, attitudes toward online exercise, and expectations of participation in exercise. A cluster analysis was used to categorize participants into groups with high and low attitude scores. Differences between high and low groups’ scores in their intention to exercise online were tested with an independent sample *t*-test. Finally, demographic differences (gender and age groups) were tested with an independent sample *t*-test and an ANOVA. Statistical analysis was conducted using SPSS version 21.

## 4. Results

### 4.1. Intention to Participate

More than half of the patients expressed a clear positive intention to participate in online exercise (54.5%) (Table 3). Those who were skeptical about it reported “safety” (54%) as the main barrier to their participation. Significantly, most of the patients (63%) believed that online exercise could be safe if the appropriate conditions were provided (Table 3). However, 54% of the sample reported that they would like to monitor all their vital health signs online—their heart rate, blood pressure, electrocardiogram, oxygen saturation, and breath rate. These are the main features that should be included in a mobile application.

### 4.2. Attitudes towards Online Exercise

In terms of their attitudes towards online exercise, the mean scores of both the affective and cognitive components were average to high (4.6 for the cognitive and 3.5 for the affective components on a 5-point scale). Regarding the cognitive dimension, the highest scores were obtained for “beneficial” (mean 4.40) and “helpful” (mean 4.30), followed by “convenient” (mean 4.28). In the affective dimensions, the highest scores were reported with the “disappointing” (mean 4.64), “stimulating” (mean 4.00) and “attractive” (mean 3.88) adjectives (Table 4).

### 4.3. Expectations of Online Exercise

Regarding the expected benefits of patients participating in online exercise, the highest scores were reported in the “improving physical condition” (mean 4.72) and “improving physical health” (mean 4.62) categories, followed by the “improving mood” (mean 4.62) and “improving psychological health” (mean 4.60) categories (Table 5).

### 4.4. Cluster Analysis

A cluster analysis was conducted to create groups with high and low attitudes and examine their intentions to participate in online programs. The Ward method using K-means clustering was used. The analysis indicated that a two-group solution was the most meaningful. This was supported by the ANOVA test. The two groups had statistically significant differences in their mean attitude scores (*p* < 0.001). The first group (N = 24) had high scores in both attitude dimensions (4.8 for the cognitive and 4.1 for the affective), while the second group (N = 26) had high scores in the cognitive dimension (4.3) and low scores in the affective dimension (2.8) (Table 6). The second group includes patients who, while they believed that online exercise was beneficial, considered it not pleasurable nor attractive.

### 4.5. Comparison between the Two Attitudes in the Groups in Terms of Intention

A *t*-test was conducted to compare the high- and low-attitude group scores in two dimensions of intention (I would like to participate, and I intend to participate). The results revealed statistically significant differences in both dimensions of intention (*t* = 2.8, *p* < 0.01 ** and *t* = 3.4, *p* < 0.001 **) (Table 7). The cluster group with high scores in both the attitude dimensions had the highest intention mean scores.

### 4.6. Comparison in Intentions to Exercise in Terms of the Age and Gender Groups

An ANOVA test was conducted to compare the two intention items in terms of the age groups. The results from the Scheff post hoc analysis revealed statistically significant differences in Intention 2 items (F = 4.8, *p* < 0.005). The oldest group had statistically significantly lower scores than all the other groups (Table 8).

A *t*-test was conducted to compare male and female scores in the two intention items. The results revealed statistically significant differences only in the Intention 2 item (*t* = 1.9, *p* < 0.05 *), with the males having statistically significantly higher scores than females (Table 9).

## 5. Discussion

This paper aimed, for the first time, to examine the attitudes of patients with CVD towards online exercise and to develop a mobile application that would be useful for doctors and physicians to check their vital health signs when exercising. As previously noted, it has been proposed that patients with CVD should avoid crowded sites in fitness and health clubs, especially in the cases of pandemics, such as COVID-19, or in the cases of flu. Exercising in their home environment gives them the chance to overcome several psychological constraints, which are perceived in a social exercise environment. Moreover, online exercise can give them the chance to exercise under the distant supervision of health professionals together with the operation of a mobile application. However, it is also well documented today [19] that, while online exercise has several health-related benefits for participants, it is also not perceived as fun and motivating when compared with the on-site exercise; this can be one of the demotivating factors for patients to involve themselves in online programs.

The results of this study first revealed that half of the patients expressed an intention to participate in online exercise, which is an encouraging finding and shows the existence of a latent demand. This is an encouraging finding for adopting technology in exercise, both in the settings of the home and in organized programs. This should be considered by exercise leaders and policymakers. Intention, however, does not always predict actual behavior [18]. These results therefore should be considered with caution. As expected, many patients were skeptical about the safety of online exercise. The use of a mobile application can, however, give them the feeling of safety if this application monitors their vital health signs such as heart rate and blood pressure when exercising. It seems that such an application can be a motivating factor for them to participate in online exercise. Safe protocols should therefore be developed in order for the programs to be perceived as appropriate at an individual level. Training sessions for patients are also required to be able to follow exercise professionals’ delivery of sessions. Close consultation with their health professionals could give them the confidence to participate in the programs. Additionally, rehabilitation centers should invest in such programs both in terms of human resources—specialized staff—but also in terms of the technology required.

According to the general attitude theory [18], both the cognitive and affective elements of attitudes should be built for any behavior to be influenced. The development of cognitive attitudes requires the distribution of information about the benefits of exercise participation for perspective participants to make a justified decision to participate. On the other hand, addressing the affective component requires positive experiences and enjoyment on the side of the participant. In this study, it was shown that almost all the patients had positive cognitive attitudes toward online exercise, which means that they did believe in the physical and psychological benefits of online exercise. They had, in some way, been convinced that, if they participated in such programs, it would help their health. However, the cluster analysis showed that one of the two attitude groups had low scores in the affective component of attitudes, which means that these patients did not believe that online exercise would be fun, pleasurable, and exciting. The analysis also showed that these low positive affective attitudes can, to some degree, determine patients’ intentions to participate in online exercise programs, even if a mobile application for monitoring their vital signs is offered. There is a clear challenge here for health and fitness professionals. Specialized and trained exercise instructors should work in cooperation with health professionals to develop appropriate exercise programs that are attractive for patients and also follow specific health protocols, which would make them safe for patients.

The results also revealed some demographic variations. As expected, older patients had significantly lower scores in their behavioral intention items than the younger groups. These results support previous studies that included mainstream populations as a sample [20]. Females also had significantly lower scores than males, which shows that female patients might be a more demanding group than male patients. In terms of patients’ expectations of online exercise, the results revealed high mean scores in the physical and psychological health items, which were related to physical conditioning and mood improvement but also physical appearance and energy regeneration. Similar expectations have been reported in studies that included the mainstream population [21].

These results have practical implications. They first show that online exercise can be considered a low-cost exercise method by health professionals and rehabilitation centers to organize and deliver appropriate programs. Patients, however, need to be first convinced that these programs are safe and that they are enjoyable. Close cooperation between health and exercise professionals is required to develop safe protocols. On the other hand, rehabilitation centers should consider investing in online technology to find alternative exercise delivery methods.

In conclusion, this study showed that online exercise can be a valuable tool to convince patients with CVD to have a more active lifestyle if it is delivered in a fun and safe manner. The use of technology can help in this direction. The results showed that patients, to a large degree, would feel safer exercising under the distant supervision of health professionals if their vital health signs were monitored online.

## 6. Study Limitations and Future Research

As previously noted, this study measured patients’ attitudes toward online exercise. According to attitude theory, behavioral intentions strongly correlate with actual behavior (exercise or not), but they do not entirely predict it [18]. It is, therefore, important in future studies to measure actual behavior by following patients’ actual behavioral patterns. The results also showed that patients would feel safer to exercise under the distant supervision of health professionals. These perceptions need to be confirmed in a real-life situation with the use of a mobile application designed for this purpose. A study with an experimental design would help us examine the real influence of such an application, as a motivating but also controlling factor. A final note should be made about the attributes, design, and delivery of online programs. These programs should be, in a way, standardized to meet certain health protocols and also be fun at the same time. More research is required to propose and develop such exercise sessions.

## Figures and Tables

**Table 1:** Demographic characteristics of the sample.

Gender Groups	Age Groups	Occupation		
Males 72%	18–25	10%	Private Sector	10%
Females 28%	26–35	36%	Public Sector	44%
	36–45	20%	Entrepreneur	18%
	46–65	34%	Housewife	8%
			University student	10%
			Other	10%

**Table 2:** Medical profile.

Sufferers of Chronic Diseases	Under Medication
Yes 16%	Yes 16%
No 84%	No 84%

**Table 3:** Intention to participate regarding the perception of safety in online exercise.

Intention to Participate		Safe	
Yes	54.5%	Yes	63.6%
Maybe	27.2%	No	36.4%
No	18.18%		

**Table 4:** Mean and SD scores of the scales.

Cognitive Items	Mean	SD
Beneficial	4.40	0.63
Helpful	4.30	0.61
Convenient	4.28	0.75
“Useful”	3.46	0.81
**Affective Items**	**Mean**	**SD**
Disappointing	4.64	0.48
Stimulating	4.00	0.72
Attractive	3.88	0.91
Interactive	3.68	0.97
Pleasant	3.60	0.63
Interesting	3.60	1.24
Funny	3.40	0.98

**Table 5:** Mean Scores and SDs.

Cognitive Scales	Mean	SD
Improve my physical condition	4.72	0.45
Improve my physical health	4.62	0.49
Improve my mood	4.62	0.49
Improve my psychological health	4.60	0.67
Improve my appearance	4.54	0.50
Increase my energy	4.54	0.64

**Table 6:** Cluster analysis based on patients’ attitudes.

Scales	Both High (N = 24)	Cognitive High/Affective Low (N = 26)	F
Cognitive	4.85	4.37	16.1 **
Attitudes	4.13	2.84	274.9 **

** *p* < 0.001.

**Table 7:** *T*-test for the comparison between the two attitude groups in terms of intention.

Scale	Cognitive High/Affective Low (N = 26)	Both High (N = 24)	
I would like to participate	3.1	4.2	*t* = 2.8, *p* < 0.01 **
I intend to participate	3.3	4.1	*t* = 3.4, *p* < 0.001 ***

*** 0.001 level, ** 0.01 level.

**Table 8:** ANOVA for the comparison of intention in terms of age.

Scale	Age 1 (18–25) (N = 5)	Age 2 (26–35) (N = 18)	Age 3 (35–45) (N = 10)	Age 4 (45–65) (N = 17)	F
	Mean				
Intention 1	4.0	3.6	4.0	3.6	non-significant
Intention 2	5.0	3.6	4.5	2.8	4.8, *p* < 0.005

**Table 9:** *T*-test for the comparison of intention in terms of gender.

Scale	Males (N = 36)	Females (N = 14)	
Intention 1	3.9	3.6	*T* = 0.67, ns
Intention 2	3.9	3.3	*t* = 1.9, *p* < 0.05 *

* 0.05 level.

## Data Availability

We prefer not to share our raw data publicly due to the nature of the sample (we did not ask for this in the consent form).
